# Mapping Research Trends and Collaborative Networks in Swarm Intelligence for Healthcare Through Visualization

**DOI:** 10.7759/cureus.67546

**Published:** 2024-08-22

**Authors:** Reji Kollinal, Jeena Joseph, Sneha M Kuriakose, Sabeen Govind

**Affiliations:** 1 Department of Computer Applications, BPC College, Piravom, IND; 2 Department of Computer Applications, Marian College Kuttikkanam (Autonomous), Kuttikkanam, IND; 3 Department of Computer Applications, St. Peter’s College, Kolenchery, IND; 4 Department of Computer Science, Rajagiri College of Social Sciences (Autonomous), Cochin, IND

**Keywords:** vosviewer, biblioshiny, bibliometric analysis, healthcare, swarm intelligence

## Abstract

Swarm intelligence, evolved from the self-organized behavior of social insects, has become an essential method under artificial intelligence for handling complex and dynamic issues. This study visualizes and analyzes the use of swarm intelligence in healthcare, focusing on its role in managing rising medical data complexity, optimizing diagnostic and therapeutic solutions, and supporting personalized healthcare. The analysis, based on literature from Scopus (2003-2024) using Biblioshiny and VOSviewer, reveals a strong increase in publications since 2017, with central themes around disease diagnosis, treatment optimization, medical image analysis, and real-time patient monitoring through frameworks like the Internet of Medical Things (IoMT) and swarm learning. Key findings include the identification of prolific authors, influential journals, and significant collaborative networks, with China and India emerging as major contributors. These insights underscore the multidisciplinary nature of swarm intelligence in healthcare, positioning it as a potential game-changer in medical diagnostics and patient care through collaborative and innovative research.

## Introduction and background

Swarm intelligence, inspired by the collective behavior of social insects like ants, bees, and termites demonstrates vast potential for solving complex problems in artificial intelligence. Its decentralized, self-organized nature and ability to adapt renders it particularly effective for dynamic, intricate issues across many domains including healthcare. The application of swarm intelligence within healthcare has gained significant momentum attributable to ever-increasing medical data complexity, imperative demand for efficient diagnostics and therapies, and personalized care requirements.

Swarm intelligence algorithms have proven remarkably useful for disease diagnosis and treatment optimization facilitating mining and analysis of substantial healthcare information to meaningfully improve major disease prognoses such as cancer, heart conditions, and tumors. For example, as highlighted by Nayar et al., swarm intelligence plays an indispensable role in diagnosis and therapies through integrated data mining techniques [1]. Moreover, Rosenberg et al. illustrated that small groups of networked radiologists functioning as a living swarm intelligence system decreased diagnostic errors by 33% while outperforming state-of-the-art deep learning when evaluating pneumonia from chest X-rays [2]. Particle swarm optimization (PSO) techniques and ant colony optimization have also demonstrated efficacy in medical imaging evaluation and disease monitoring serving to enhance diagnostic processes and outbreak surveillance. Simran and Singh offer a thorough review of particle swarm optimization - ant colony optimization (PSO-ACO) optimization and swarm intelligence applications within medical imaging and disease tracking emphasizing their potential to transform healthcare [3].

Swarm intelligence frameworks, like the Internet of Medical Things (IoMT), enable real-time continuous and remote patient monitoring while supporting essential decision-making processes in healthcare. These frameworks are particularly valuable for smart healthcare environments, delivering ceaseless tracking of patients, especially seniors, to boost care provision and outcomes. El-Shafeiy and Abohany debate the strength of a swarm intelligence system in IoMT for intelligent healthcare infrastructure [4]. In addition, swarm learning combines edge computation and blockchain to safeguard data privacy while improving the precision of medical classifiers. This hybrid approach facilitates the joining of decentralized data pools, enhancing analysis and remedy paths. Warnat-Herresthal et al. demonstrate the practicability of employing swarm learning for evolving disease classifiers, displaying its potential to quicken customized medication [5]. Hybrid swarm cleverness algorithms have since been advanced to boost the performance of care services, particularly in streamlining task scheduling and asset distribution in cloud computing platforms. Hassan et al. propose a mixed optimization algorithm that merges PSO and the salp swarm algorithm (SSA), exhibiting its superiority over present algorithms regarding makespan, waiting time, and resource use [6].

Recent advancements in swarm intelligence have also shown promise in optimizing drug delivery systems, bioinformatics, and personalized medicine. For instance, hybrid swarm intelligence algorithms have been employed to optimize nanoparticle delivery systems, enhancing the precision and efficacy of targeted drug delivery [7]. In bioinformatics, swarm algorithms such as artificial bee colony (ABC) have been utilized for protein structure prediction, improving the accuracy and speed of computational biology processes [8]. Additionally, swarm intelligence has been integrated with machine learning to develop predictive models for personalized medicine, which tailor treatments based on individual genetic profiles [9]. Swarm-based optimization has also been applied to enhance the performance of wearable medical devices, ensuring better monitoring and management of chronic diseases [10]. Furthermore, swarm intelligence is being used to design more efficient and adaptive healthcare logistics systems, which streamline the supply chain and improve the delivery of medical supplies [11].

In a recent study, Bijli et al. underscored the very dynamic domain of swarm nanorobotics, boasting its disruptive technologies to come for health applications, from oncology and drug delivery to surgery. Though much hope is placed on the promising abilities it does carry, there are challenges of formidable dimensions in regard to swarm control, navigation, and obstacle avoidance if nanorobots are supposed to envelop seamless integration into medical practice [12]. Kioskli et al. proposed a novel framework inspired by swarm intelligence for improving cybersecurity in healthcare ICT, which imitates biological swarm behavior to improve threat detection, analysis, and response. This approach brings together such advanced technologies as artificial intelligence, security engineering, and privacy engineering to create a highly interconnected system supporting dynamic intelligence, scalability, and fault tolerance within healthcare infrastructures, hence strengthening the resilience of digital healthcare systems and supply chains against attacks [13].

Bibliometric analysis provides quantitative insights into scientific publications through computer tools, assessing core authors and key findings while revealing interconnected relationships [14]. This statistical approach offers perspectives on productivity and influence by evaluating journal impact factors by analyzing interlinks between works [15,16]. Data sourced from databases like Web of Science, Scopus, or Google Scholar undergoes cleaning to remove duplicates prior to an in-depth examination. Citation and collaboration techniques discern research clusters and networking, rendering bibliometric methods exceptionally beneficial for researchers, administrators, and librarians by facilitating efficient navigation amid vast scientific literature [17,18].

RStudio provides a robust platform for developing in R, offering desktop and web-based interfaces alike [19]. Bibliometrix, an open-source R package, facilitates visual exploration of scholarly literature through the import of references from databases, metric calculation, and representation of datasets for diverse investigations [20,21]. VOSviewer is analytical software permitting visualization and analysis of bibliographic networks, generating and portraying co-author, co-citation, and keyword co-occurrence mappings. With intuitive design and customizable graphics, VOSviewer aids in uncovering hidden patterns within publication repositories, revealing complex relationships and evolving trends [22,23]. Its strong functionality combined with usability earns it widespread application across disciplinary bibliometric inquiries.

This study aims to visualize research trends and collaborative networks within the emerging field of swarm intelligence applied to healthcare. The review maps the evolution of swarm-based methodologies that were employed in different medical scenarios through the systematic analysis of scientific publications from 2003 to 2024. It involves the identification of seminal works, key authors, and primary journals that make a significant impact in the domain, unearthing dominant topics, technologies, and areas of application. These would especially be algorithms of major importance, such as PSO and ACO, which help in the enhancement of diagnostic accuracy, optimization of treatment plans, and improvement of patient monitoring. This focused review of development and collaboration in this interdisciplinary field is streamlined by targeting these core methodologies and their direct applications to healthcare.

## Review

Methods

The literature search encompassed publications retrievable from the Scopus digital library as of July 1, 2024, using the search terms "Swarm Intelligence" AND "Health" to identify relevant works published within the designated period without language restrictions. Initially, all document types, including articles, conference papers, and book chapters, were considered to ensure comprehensive coverage. The final dataset was refined to include only those works that substantively engaged with swarm intelligence within medical contexts. Non-original materials such as letters, reviews, and bibliographies lacking critical indexing metadata were excluded to focus exclusively on primary research contributions. The retrieved records were meticulously reviewed for data integrity and relevance; duplicates were removed based on title, authorship, and publication details. Non-research documents, such as editorials and technical notes, were also excluded. Additionally, documents with incomplete metadata or those not directly relevant to the intersection of swarm intelligence and healthcare were excluded. The cleaned dataset was organized in comma-separated values (CSV) format and analyzed using Biblioshiny and VOSviewer software, ensuring the study's transparency and reproducibility by providing a comprehensive and accurate representation of the literature. Table 1 highlights the main aspects of the articles included in the final analysis, providing an overview of the key characteristics of the selected studies.

**Table 1:** Main aspects of the articles The dataset spans from 2003 to 2024 and includes various sources such as journals, books, and conference proceedings. It comprises a total number of documents analyzed, with the annual growth rate expressed as a percentage. The average age of the documents, average citations per document, and total references are detailed. The table also includes the number of unique keywords generated by the algorithm (keywords plus) and those provided by authors (author's keywords). Additionally, it highlights the total number of individual authors, single-authored documents, co-authors per document, and the percentage of international co-authorships. The table further breaks down the number of journal articles, book chapters, and conference papers included in the dataset.

Description	Results
Main information about data	
Timespan	2003:2024
Sources (journals, books, etc.)	313
Documents	455
Annual growth rate %	19.61
Document average age	4.2
Average citations per doc	14.46
References	15576
Document contents	
Keywords plus (ID)	3725
Author's keywords (DE)	1378
Authors	
Authors	1481
Authors of single-authored docs	16
Authors collaboration	
Single-authored docs	16
Co-authors per doc	4
International co-authorships %	26.15
Document types	
Article	245
Book chapter	22
Conference paper	188

Annual scientific production

**Figure 1:**
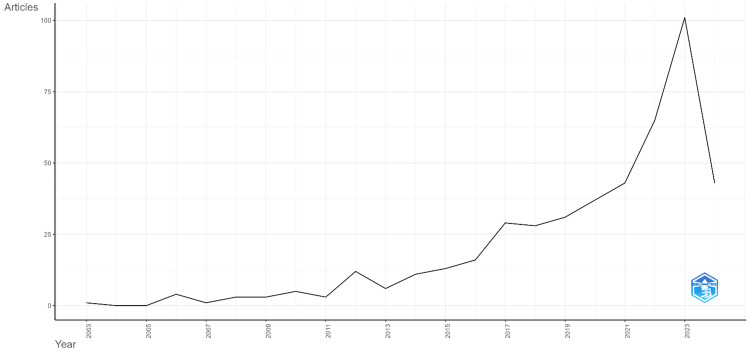
The annual scientific production This line graph illustrates the trend of scientific article production from 2003 to 2024. The X-axis, labeled "Year," denotes the timeline of the scientific production, while the Y-axis, labeled "Articles," indicates the quantity of scientific publications. The graph shows a noticeable increase in the number of articles published annually, with a significant spike in recent years, highlighting the growing scientific output over the analyzed period.

Figure 1 illustrates the annual scientific production in swarm intelligence applied to healthcare from 2003 to 2024. The data shows a gradual increase in the number of publications over the years, with a notable surge beginning around 2017. This upward trend indicates a growing interest and recognition of the potential of swarm intelligence in the healthcare sector. The peak in scientific production occurred in 2023, with over 100 articles published, reflecting a significant focus on this interdisciplinary field during that year. However, there is a noticeable decline in the number of publications in 2024. This pattern suggests that while the field experienced a period of rapid growth, it may be stabilizing or adjusting after the peak in 2023. The overall trend underscores the increasing importance of swarm intelligence in healthcare research and its evolving nature over the past two decades. The decrease in publications in 2024, following the 2023 peak, may result from several factors, such as a period of consolidation and validation after recent innovations, shifting research priorities due to emerging areas and global circumstances, and potential publication delays. This trend could influence future research directions by encouraging a focus on refining and validating existing methodologies, exploring new interdisciplinary approaches, and addressing the challenges posed by these emerging areas, ultimately driving the next wave of advancements in swarm intelligence for healthcare.

Most relevant authors

Table 2 highlights the most prolific authors in the field of swarm intelligence in healthcare. Bacanin N leads with 11 articles, indicating a substantial contribution to the research landscape. Li J, Zhang J, and Zivkovic M each have nine articles, showcasing their significant involvement and influence in advancing this interdisciplinary domain. Chen H and Chen Y have each contributed six articles, further underscoring their active roles in this field. Das S, Heidari AA, Li X, and Liu J, each with five articles, also demonstrate noteworthy participation in developing swarm intelligence applications for healthcare. The presence of these authors reflects a diverse and collaborative research community driving innovation and knowledge expansion in utilizing swarm intelligence for healthcare solutions.

**Table 2:** Most relevant authors This table lists the names of the most relevant authors in the dataset, based on the number of articles they have published from 2003 to 2024. The table highlights the contribution of each author to the body of research within the specified timeframe by indicating the number of articles published by each author. This metric underscores the significant impact and productivity of these authors in the scientific community.

Authors	Articles
Bacanin N	11
Li J	9
Zhang J	9
Zivkovic M	9
Chen H	6
Chen Y	6
Das S	5
Heidari AA	5
Li X	5
Liu J	5

Trend topics

**Figure 2:**
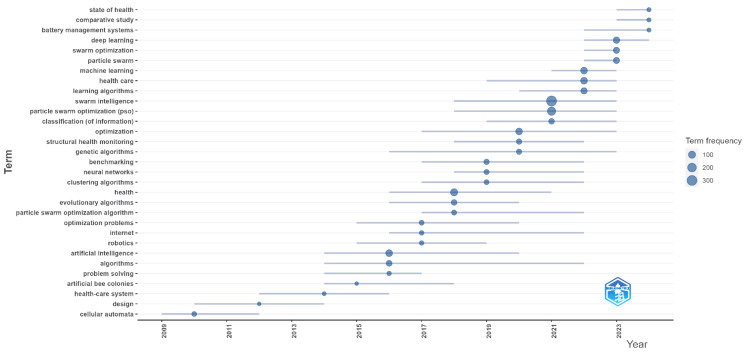
Trend topics This bubble chart displays the frequency and duration of research terms from 2006 to 2024. The X-axis, labeled "Year," represents the timeline, while the Y-axis, labeled "Term," lists frequently used research terms. The size of each bubble indicates the term frequency, with larger bubbles representing higher frequency. Horizontal lines show the duration of each term's relevance in the literature, providing insight into the persistence and prominence of specific terms over time.

Figure 2 illustrates the dynamic trends and key topics within the field of swarm intelligence as applied to healthcare, showcasing both the frequency and temporal distribution of various terms. The size of each bubble indicates the count of occurrences. "Swarm intelligence" (313) and "particle swarm optimization (PSO)" (170) are prominently featured, reflecting their central roles from 2018 to 2023. The integration of these methodologies with health topics, evidenced by terms like "health" (100), "health care" (79), and "machine learning" (79), underscores a significant surge in recent interest. Foundational terms such as "algorithms" (42) and "artificial intelligence" (81) have maintained relevance over a broader timespan, indicating their critical role. Emerging trends include "deep learning" (65) and "learning algorithms" (57), highlighting advancements in adaptive systems. Additionally, terms such as "structural health monitoring" (28) and "classification" (36) illustrate the diverse applications of swarm intelligence. Overall, these trends depict a comprehensive and evolving research landscape where swarm intelligence, combined with advanced algorithms and methodologies, enhances healthcare solutions and outcomes, underscoring its potential to address complex challenges and improve patient care.

Three field plot

**Figure 3:**
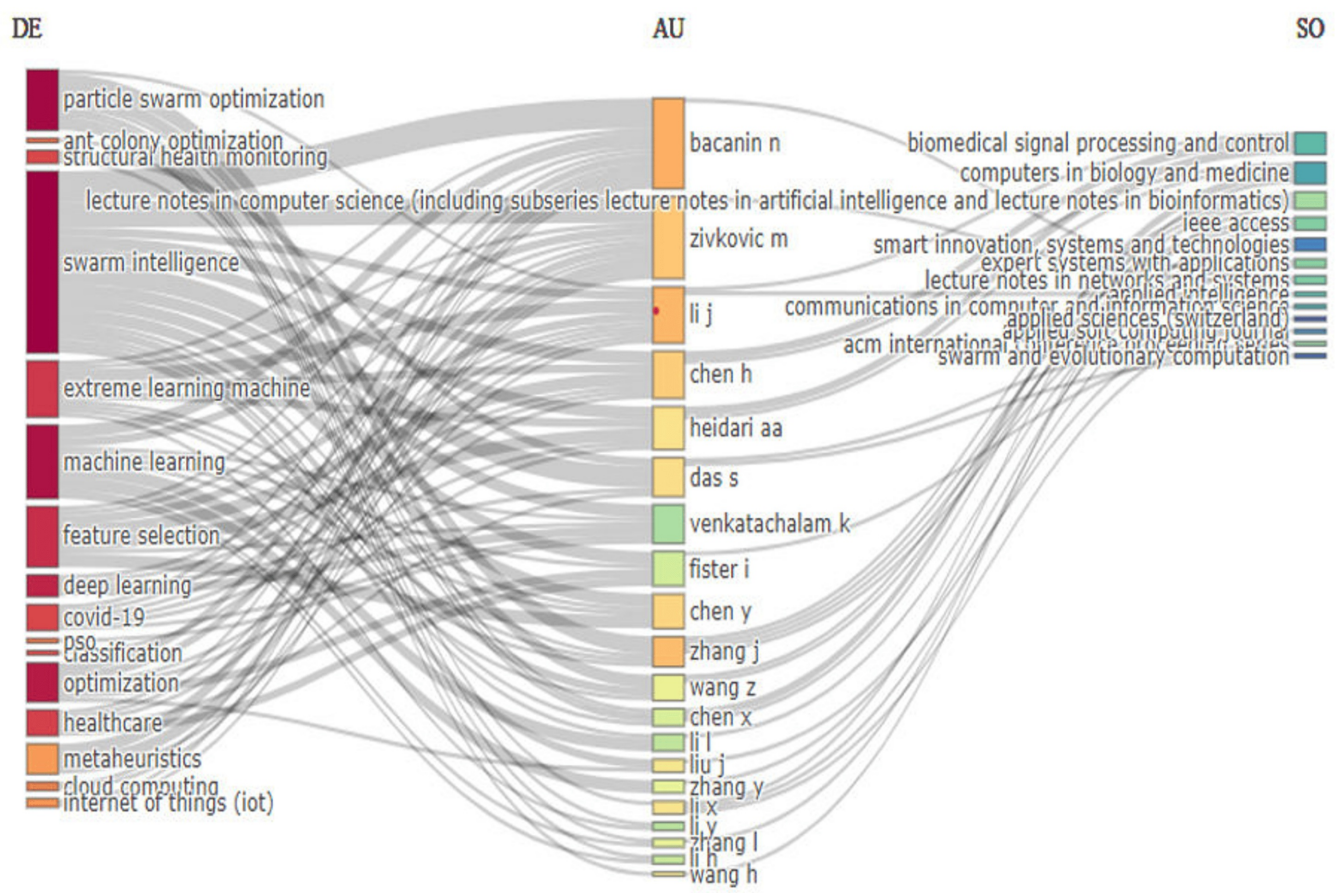
Three field plot showcasing the relationship between keywords (DE), authors (AU), and sources (SO) This diagram shows relationships between keywords, authors, and sources. The left column lists frequently used keywords (DE). The middle column lists authors (AU). The right column lists sources (SO), such as journals and conferences. Lines connect keywords to authors and authors to sources, illustrating the associations between them.

The three-field plot in Figure 3 provides a comprehensive visualization of the intricate relationships between keywords (DE), authors (AU), and sources (SO) in the domain of swarm intelligence applied to healthcare. This plot serves as a valuable tool for understanding how different elements of research interconnect and where the most significant contributions are concentrated. Prominent keywords such as "particle swarm optimization," "swarm intelligence," "extreme learning machine," "machine learning," "deep learning," "optimization," "healthcare," and "IoT" dominate the field. These keywords represent the core themes around which the research in swarm intelligence and healthcare revolves. For instance, the frequent occurrence of "particle swarm optimization" and "machine learning" highlights the importance of these methodologies in solving complex healthcare problems, from medical imaging to predictive analytics. The presence of "IoT" and "deep learning" as emerging keywords suggests a growing integration of advanced technologies with swarm intelligence, pointing toward future directions in research.

Key authors such as Bacanin N, Li J, Zivkovic M, Chen H, Heidari AA, Das S, Venkatachalam K, Fister I, Chen Y, Zhang J, Li X, Zhang Y, and Wang H are linked to multiple keywords, indicating their extensive contributions across various aspects of swarm intelligence in healthcare. The connections between these authors and multiple keywords reflect their interdisciplinary work and significant influence in advancing the field. For example, Bacanin N’s association with keywords like "particle swarm optimization" and "extreme learning machine" underscores his pivotal role in applying these techniques to healthcare challenges. Similarly, other authors are seen contributing across a range of topics, demonstrating the collaborative and multifaceted nature of this research area. The plot also highlights important publication sources, including "Biomedical Signal Processing and Control," "Computers in Biology and Medicine," "Lecture Notes in Computer Science," "Expert Systems with Applications," "IEEE Access," and "Swarm and Evolutionary Computation." These journals and conference proceedings are the primary venues for disseminating significant findings in the field. The presence of these sources in the plot underscores their role in fostering research on swarm intelligence and its applications in healthcare. For instance, the frequent appearance of "Biomedical Signal Processing and Control" suggests its importance in publishing research related to the signal processing applications of swarm intelligence in medical settings.

In summary, the three-field plot not only maps out the landscape of swarm intelligence research in healthcare but also provides actionable insights into the key players, topics, and publication venues driving this dynamic and interdisciplinary field. The dense interconnections between authors, keywords, and sources reveal the interdisciplinary and collaborative nature of swarm intelligence research in healthcare, which is essential for driving innovation in this area. Additionally, the plot uncovers emerging research trends, particularly the integration of swarm intelligence with advanced technologies like deep learning and IoT, indicating the likely future direction of research. Last, by identifying the most prolific authors and their associated keywords, the plot highlights influential contributors and key publication venues, offering valuable guidance for new researchers in the field.

Co-occurrence of keywords

**Figure 4:**
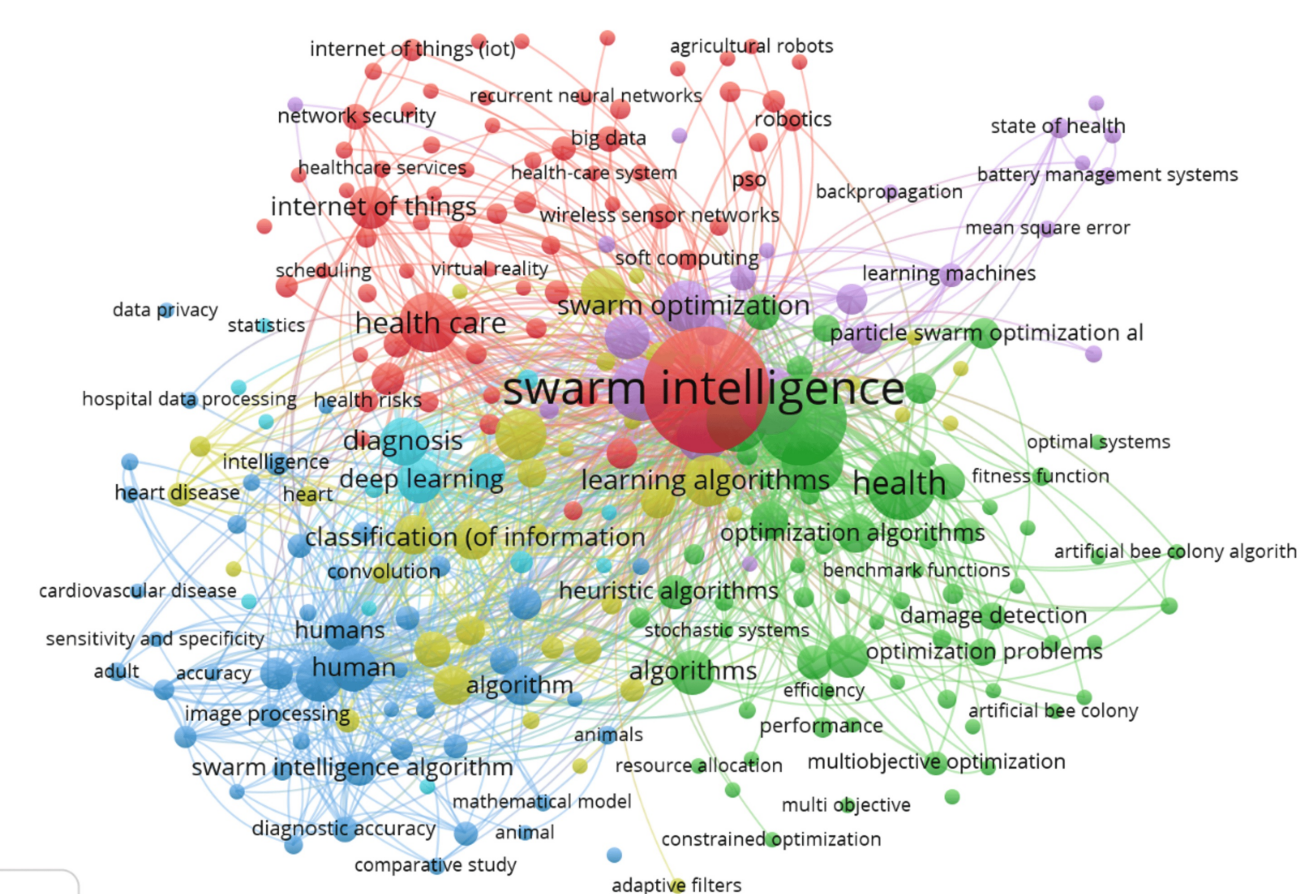
Co-occurrence of keywords This network visualization, generated using VOSviewer, shows the co-occurrence of keywords in the dataset. Each node represents a keyword, with node size reflecting the keyword's frequency. Edges indicate co-occurrence, with thicker lines showing stronger relationships. Different colors group keywords into clusters, representing related topics or themes.

Figure 4 presents the co-occurrence network of keywords in the field of swarm intelligence applied to healthcare, created using VOSviewer. This visualization highlights the interconnectedness and frequency of various research themes, with larger nodes representing more frequently occurring keywords. The central theme of "swarm intelligence" is the most prominent node, indicating its fundamental role and extensive connections to related terms. "Swarm optimization" is another significant node, underscoring its widespread application. The network reveals several key clusters: one centered around "health" and "healthcare," with closely related terms such as "diagnosis" and "deep learning," reflecting the integration of swarm intelligence in medical diagnostics and treatment. Other important clusters include "learning algorithms," "optimization algorithms," and "machine learning," highlighting the synergy between swarm intelligence and various computational techniques. Additionally, terms like "internet of things (IoT)," "robotics," and "security" form clusters, showing the diverse applications of swarm intelligence in healthcare technology and systems. Overall, the co-occurrence network illustrates a rich and interconnected research landscape, emphasizing the multidisciplinary nature of swarm intelligence in advancing healthcare solutions.

Co-authorship between countries

**Figure 5:**
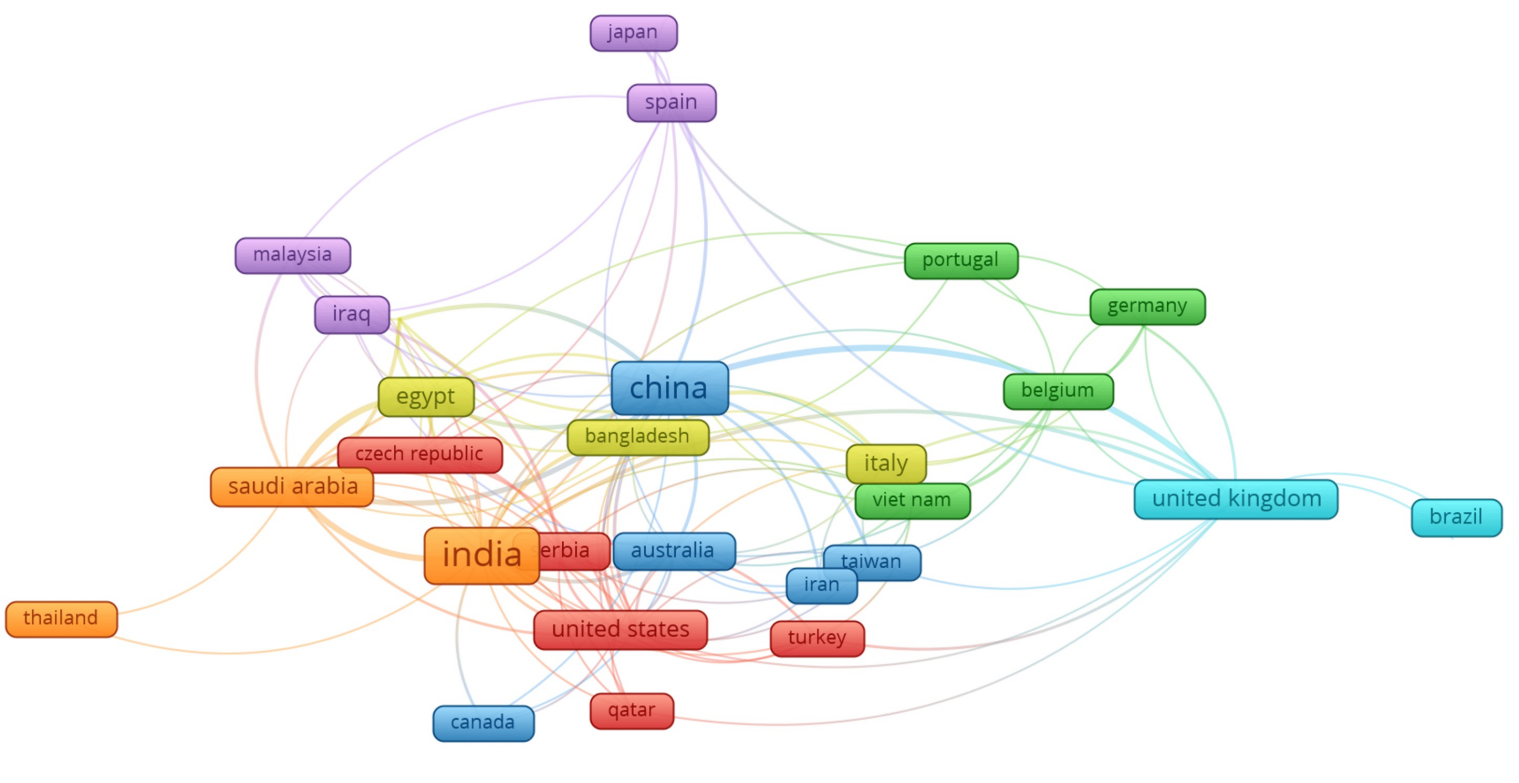
Co-authorship of countries This network visualization, generated using VOSviewer, depicts co-authorship relationships between countries. Each node represents a country, with node size reflecting the number of international co-authorship publications. Edges indicate co-authorship relationships, with thicker lines showing stronger collaborative ties. Different colors group countries into clusters, representing regions or networks that frequently collaborate.

Figure 5 illustrates the co-authorship relationships between countries in the field of swarm intelligence applied to healthcare, showcasing the collaborative nature of international research efforts. China, represented by the largest node, emerges as a central hub with significant contributions and numerous collaborations with other countries. India is also a prominent node, demonstrating extensive collaboration, particularly with Saudi Arabia, Egypt, and the United States. The United States is a major player in the network, with strong connections to China, India, and Canada. The United Kingdom, another important node, has numerous collaborative links, especially with European countries like Germany, Belgium, and Portugal. Italy shows notable connections within Europe, collaborating extensively with Germany, Belgium, and Portugal, and also linking to China and Vietnam. Other countries with significant collaboration patterns include Germany, actively partnering with neighboring European countries and the United States, Egypt, collaborating with countries in both Africa and Asia, and Saudi Arabia, engaging in research partnerships with India, Egypt, and China. This visualization underscores the global nature of research in swarm intelligence applied to healthcare, with dense interconnections reflecting a high degree of international collaboration. These networks facilitate the sharing of knowledge, resources, and expertise, driving forward advancements in this interdisciplinary field and highlighting the importance of collaborative efforts in addressing complex healthcare challenges through swarm intelligence.

Discussion

The application of swarm intelligence in healthcare has demonstrated substantial promise, with a significant increase in research output over the past two decades. This upward trend highlights the growing recognition of swarm intelligence's potential to address complex healthcare challenges. Key algorithms such as PSO and ant colony optimization (ACO) have been particularly effective in medical image analysis, disease surveillance, and optimization tasks, underscoring their versatility and impact on healthcare solutions. Emerging trends identified through bibliometric analysis reveal a strong integration of swarm intelligence with advanced technologies like deep learning and the Internet of Things (IoT). This integration has led to the development of sophisticated diagnostic tools and real-time patient monitoring systems, which are crucial for enhancing healthcare delivery and outcomes. The surge in interest and applications of swarm intelligence in healthcare, peaking in scientific production in 2023, underscores the field's dynamic nature. However, the decline in publications in 2024 suggests a potential stabilization or shift in research focus, indicating that the field may be entering a phase of consolidation or exploring new directions.

Key contributions from prolific authors such as Bacanin N, Li J, and Zhang J have significantly advanced the field, showcasing the interdisciplinary and collaborative nature of swarm intelligence research in healthcare. Their work, often published in high-impact journals, highlights the broad applicability of these methodologies and their potential to drive innovation in healthcare. The bibliometric analysis also reveals the diverse and global nature of research in this field, with significant contributions from countries like China, India, the United States, and various European nations. The strong international collaborations reflected in co-authorship networks highlight the global effort to leverage swarm intelligence for healthcare advancements. In conclusion, the application of swarm intelligence in healthcare has made significant strides, demonstrating its potential to improve diagnostic accuracy, treatment optimization, and personalized medicine. The continued exploration and integration of advanced technologies with swarm intelligence will likely drive further innovations and improvements in patient care. The global and collaborative research efforts in this field underscore the importance of shared knowledge and resources in addressing complex healthcare challenges through swarm intelligence.

Implications of emerging trends

Swarm intelligence integrated with deep learning, IoT, and blockchain is going to fundamentally shift healthcare research and application. As time goes on, the trends will engender more sophisticated and adaptive healthcare solutions in percentage terms. For instance, swarm learning enables the interplay with decentralized data processing systems that allow the realization of robust models capable of executing diagnosis and real-time disease prediction in areas with low resources. This would greatly improve global health equity, making cutting-edge diagnostic tools available to a wide spectrum of populations. The development of further hybrid swarm intelligence algorithms that include a variety of optimization techniques would have to be refactored to deal with the growing complexity of health data. Further research will be directed toward optimizing such hybrid models to apply even more widely in genomics, personalized medicine, and telemedicine, therefore going beyond what seems possible to achieve with swarm intelligence in healthcare.

Ethical considerations

The major ethical challenges that will accrue from swarm intelligence technologies as they become integrated into the healthcare system will mainly be around data privacy, security, and algorithmic transparency. A major concern may be the decentralized nature of the swarm intelligence, where it manages large-scale data; this might compromise the secrecy and integrity of sensitive health information. To build trust from the users and other stakeholders, it will be very important to ensure that such systems have strong privacy-preserving features, like secure multiparty computation and differential privacy. In addition, opacity, the characteristic of most swarm algorithms, leads to complexities and presents great difficulties in being able to interpret and explain the decision-making processes in potential critical healthcare scenarios. Such risks should be reduced by future research into transparent and interpretable swarm models that allow a healthcare provider to trace and justify the results of an algorithmic decision. Addressing these ethical considerations will help the healthcare industry proceed with care through the challenges brought about by the widespread adoption of swarm intelligence and guarantee the reaping of its benefits without selling outpatient rights and safety.

Research gaps

Despite significant advancements, several research gaps remain in the application of swarm intelligence in healthcare. One key gap is the need for more robust and scalable algorithms capable of handling the vast and heterogeneous nature of healthcare data. Current algorithms often struggle with real-time data processing and integration from multiple sources, such as electronic health records, wearable devices, and genomic data. Another area needing further exploration is the validation and standardization of swarm intelligence models across different healthcare settings and populations. The diversity in healthcare practices and patient demographics necessitates algorithms that are adaptable and generalizable. Additionally, ethical considerations related to data privacy, security, and algorithmic transparency must be addressed to foster trust and widespread adoption in clinical environments. Addressing these gaps through interdisciplinary collaboration and innovative methodologies will enhance the effectiveness and reliability of swarm intelligence applications in healthcare.

Practical implications

The practical implications of swarm intelligence in healthcare are profound, offering potential improvements in diagnostic accuracy, treatment optimization, and personalized medicine. By leveraging swarm intelligence, healthcare providers can develop more accurate predictive models for disease diagnosis and prognosis, enhancing patient outcomes. Real-time patient monitoring and decision support systems, powered by swarm intelligence, can lead to more responsive and efficient healthcare delivery, particularly in critical care and emergency settings. Moreover, the integration of swarm intelligence with other technologies, such as IoT and blockchain, can facilitate secure and efficient data sharing across healthcare networks, improving coordination and continuity of care. Embracing these practical applications can drive innovation in healthcare, ultimately leading to better patient care and resource management.

Limitations

This study primarily relied on publications retrieved from the Scopus database, which, although comprehensive, may not capture all relevant research, particularly those published in less mainstream journals. Additionally, given the rapidly evolving nature of swarm intelligence in healthcare, new developments could shift the landscape beyond what is reflected in this analysis. Furthermore, the bibliometric approach used in this study focuses more on quantitative measures, such as publication and citation counts, which may not fully capture the qualitative aspects or the practical impact of certain research contributions. While these factors may affect the comprehensiveness of the findings, the study nonetheless offers valuable insights into the current state of research and collaboration within this emerging field.

## Conclusions

The application of swarm intelligence in healthcare represents a burgeoning field with the potential to revolutionize medical diagnostics, treatment, and patient management. Through a comprehensive bibliometric analysis, this study highlights the growing body of research and the collaborative networks that underpin advancements in this domain. The findings underscore the interdisciplinary nature of swarm intelligence research and its increasing relevance in addressing complex healthcare challenges. However, to fully realize the benefits of swarm intelligence, it is imperative to address existing research gaps and ethical concerns. Future research should focus on developing scalable and adaptable algorithms, ensuring model validation across diverse settings, and prioritizing data privacy and security. By continuing to explore and refine swarm intelligence applications, the healthcare industry can move closer to achieving more accurate, efficient, and personalized care for patients worldwide.

## References

[1] Nayar N, Ahuja S, Jain S (2019). Swarm intelligence and data mining: a review of literature and applications in healthcare. Proc Third Int Conf Adv Inform Comput Res.

[2] Rosenberg L, Lungren M, Halabi S (2018). Artificial swarm intelligence employed to amplify diagnostic accuracy in radiology. 2018 IEEE IEMCON.

[3] Simran Simran, Singh J (2023). A comprehensive survey of PSO-ACO optimization and swarm intelligence in healthcare: implications for medical image analysis and disease surveillance. 2023 ASIANCON.

[4] El-Shafeiy E, Abohany A (2020). A new swarm intelligence framework for the Internet of Medical Things system in healthcare. Swarm Intelligence for Resource Management in Internet of Things.

[5] Warnat-Herresthal S, Schultze H, Shastry KL (2021). Swarm Learning for decentralized and confidential clinical machine learning. Nature.

[6] Hassan K, Abdo A, Yakoub A (2022). Enhancement of health care services based on cloud computing in IoT environment using hybrid swarm intelligence. IEEE Access.

[7] Wang L, Gan C, Sun H, Feng L (2023). Magnetic nanoparticle swarm with upstream motility and peritumor blood vessel crossing ability. Nanoscale.

[8] Karaboga D, Akay B, Karaboga N, Pham D (2020). A survey on the studies employing machine learning (ML) for enhancing artificial bee colony (ABC) optimization algorithm. Cogent Eng.

[9] Ahmed Z, Mohamed K, Zeeshan S, Dong X (2020). Artificial intelligence with multi-functional machine learning platform development for better healthcare and precision medicine. Database (Oxford).

[10] El-shafeiy E, Sallam KM, Chakrabortty RK, Abohany AA (2021). A clustering based Swarm Intelligence optimization technique for the Internet of Medical Things. Expert Syst Appl.

[11] Zhao G (2023). Application of swarm intelligence optimization algorithm in logistics delivery path optimization under the background of big data. J Funct Spaces.

[12] Bijli MK, Verma P, Singh AP (2024). A systematic review on the potency of swarm intelligent nanorobots in the medical field. Swarm Evol Comput.

[13] Kioskli K, Papastergiou S, Fotis T, Silvestri S, Mouratidis H (2024). A self-organized swarm intelligence solution for healthcare ICT security. 15th Int Conf Appl Hum Factors Ergon.

[14] Calof J, Søilen KS, Klavans R, Abdulkader B, Moudni IE (2022). Understanding the structure, characteristics, and future of collective intelligence using local and global bibliometric analyses. Technol Forecast Soc Change.

[15] do Carmo G, Felizardo LF, de Castro Alcântara V, da Silva CA, do Prado JW (2023). The impact of Jürgen Habermas's scientific production: a scientometric review. Scientometrics.

[16] Hajkowicz S, Sanderson C, Karimi S, Bratanova A, Naughtin C (2023). Artificial intelligence adoption in the physical sciences, natural sciences, life sciences, social sciences and the arts and humanities: a bibliometric analysis of research publications from 1960-2021. Technol Soc.

[17] Donthu N, Kumar S, Mukherjee D, Pandey N, Lim WM (2021). How to conduct a bibliometric analysis: an overview and guidelines. J Bus Res.

[18] Joseph J, Thomas B, Jose J, Pathak N (2024). Decoding the growth of multimodal learning: a bibliometric exploration of its impact and influence. Int Dec Tech.

[19] Racine J (2012). RStudio: a platform-independent IDE for R and Sweave. J Appl Econ.

[20] Thomas B, Joseph J, Jose J (2023). Explorative bibliometric study of medical image analysis: unveiling trends and advancements. Sci Vis.

[21] Savita Savita, Verma N (2020). A review study on big data analysis using R Studio. Int J Eng Technol Manag Res.

[22] Agbo FJ, Oyelere SS, Suhonen J, Tukiainen M (2021). Scientific production and thematic breakthroughs in smart learning environments: a bibliometric analysis. Smart Learn Environ.

[23] van Eck NJ, Waltman L (2010). Software survey: VOSviewer, a computer program for bibliometric mapping. Scientometrics.

